# Horizon Scanning Methods for Health Care Technology Innovation Identification: Rapid Scoping Review of Patent Research Studies

**DOI:** 10.2196/70323

**Published:** 2025-09-11

**Authors:** Sonia Garcia Gonzalez-Moral, Erin Pennock, Olushola Ewedairo, Elizabeth Green, James Elgey, Andrew Mkwashi

**Affiliations:** 1 NIHR Innovation Observatory, Population Health Sciences Institute, Newcastle University, The Catalyst Room 3.12, 3 Science Square Newcastle Helix, Newcastle upon Tyne, NE4 5TG, United Kingdom, 44 0191 2082262; 2 Center for Cancer, Population Health Sciences Insitute, Newcastle University, Newcastle upon Tyne, United Kingdom

**Keywords:** horizon scanning, patent analysis, medtech, rapid scoping review, health care innovation

## Abstract

**Background:**

Patents are an early sign of innovation, yet their role in horizon scanning for health care remains unclear.

**Objective:**

This study investigates the role of, and methods for, patent analysis in advancing health care technology innovation in a sector that is characterized by diverse health care technologies and significant research investment. Patents are critical early indicators of innovation, supporting horizon scanning and weak signal detection. The study aimed to identify intellectual property sources, evaluate methods for patent retrieval and analysis, and outline objectives for using patent data to anticipate trends and inform health care strategies.

**Methods:**

A rapid scoping review was conducted following Cochrane Rapid Review Methods recommendations and PRISMA (Preferred Reporting Items for Systematic Reviews and Meta-Analyses) guidelines, with a preregistered protocol on the Open Science Framework. Searches in Embase, IEEE Xplore, and Web of Science targeted records published 2020 onward to capture the most recent sources, methods, and tools. Three independent reviewers screened studies using Rayyan (Qatar Computing Research Institute). We included any study type published since 2020 that provided patent sources data, methods, and tools applied to the study of health care technologies. Our data extraction included bibliographic details, study characteristics, and methodological information. Risk of bias assessments were not undertaken. Narrative and tabular methods, supplemented by visual charts, were used to synthesize findings.

**Results:**

Our searches identified 1741 studies, of which 124 were included after title, abstract, and full-text screening, with 54% being original research, 43.5% reviews, and the remainder being conference abstracts (2.5%). Most studies (68%) relied solely on patent databases, while others searched the gray and published literature. Research objectives of the included studies were grouped into 10 themes, with trend analysis (50%) and the provision of recommendations for future research, policy, and strategy development (20%) being the most common. Our review identified up to 47 patent databases, with 27% of studies using multiple sources. Whenever time limits were reported, the mean time horizon for patent searches was 24.6 years, ranging from 1900 to 2019. Automated approaches, used in 33% (n=43) of studies, frequently used tools such as Gephi (Gephi Consortium) for network visualization. Disease mapping based on National Institute for Health and Care Excellence classification indicated that cancer (19%) and respiratory conditions (16%), particularly COVID-19, were key areas.

**Conclusions:**

Patent data are valuable for identifying technological trends and informing policy and research strategies. While patents provide crucial insights into emerging technologies, inconsistent deduplication practices across studies pose the risk of data inflation, accentuating the need for transparency and rigor. Finally, this review emphasized the importance of data transformation and visualization in detecting emerging trends, with Python and R being the most commonly used programming languages for developing custom tools.

## Introduction

According to the World Intellectual Property Office, a patent is an exclusive right granted for an invention [1]. From the moment a patent is filed, a full disclosure of the invention to a patent office would have occurred [1]. This information will be made available to the public in due course and often prior to the patent being granted [2]. In the health care technology context, patented innovations may include new pharmaceuticals, diagnostics, and medical devices. Often, inventors will apply for patents before clinical trials are initiated to protect the intellectual property of the innovative technology in the trial. However, in the case of low-risk medical devices, where clinical trials are not always conducted, patents may be one of the few indications of a new innovative product before it is introduced to the market. Therefore, patents may be considered as one of the first signs of innovation and are often searched in horizon scanning studies alone or in combination with other sources of weak signal detection [3].

However, with the complexity of some types of health care technologies such as medical devices, as well as the advent of artificial intelligence and its integration into health care technologies, it is quite difficult to identify early signs of innovation in a timely manner. Some digital applications, for instance, evolve rather rapidly, while most class 1 (low risk) medical devices do not undergo clinical trials. Yet, these technologies serve as a primary source of weak signal detection for emerging health care technologies used by the Innovation Observatory (IO), the UK national horizon scanning and research intelligence body, funded by the National Institute for Health and Care Research [4].

Furthermore, a thorough understanding of the health care technology development pathway from ideation to market is imperative to support early warning and alert systems which will, in turn, help inform strategic decision-making, the formulation of health policy, the allocation of research and development funds, and play a vital role in anticipating regulatory challenges associated with emerging technologies. Despite previous horizon scanning and forecast studies conducted in the context of the UK health care horizon scanning needs, it remains unclear how patent analysis methods are being used in forecasting technology emergence globally and how and whether current horizon scanning methods can be enhanced by the regular use of patent analysis studies [5].

Anticipating health care technology innovation before market entry is a key challenge for horizon scanning activities. However, the role of patent landscape analysis within existing horizon scanning methods remains underexplored. For instance, could routine patent analysis help identify emerging MedTech opportunities earlier, thereby enabling more proactive innovation support? Might it also inform the development of regulatory frameworks to reduce delays in technology adoption? To assess the potential of integrating patent analysis into existing horizon scanning practices, we reviewed recent literature on patent studies related to health care technologies. The study had 3 main aims: to identify key intellectual property sources, such as patent databases; to examine methods used for patent retrieval and analysis; and to determine the primary purposes for using patent analysis in this context. The findings aim to support the standardization of weak signal detection and offer a methodological foundation for integrating patent landscape analysis [6] into horizon scanning, both within the IO and among wider national and international bodies, particularly for early-stage technologies.

## Methods

### Overview

A rapid scoping review was undertaken to efficiently examine the published literature and answer the predetermined research questions on methods, tools, and purpose for patent landscape analysis [7]. We used a scoping review approach to explore the breadth of the subject area and to map the recent patent analysis methods research landscape, while also accelerating some of the traditionally more time-consuming aspects of a systematic review by embracing the recommendations for rapid reviews set out by the Cochrane Rapid Review Methods working group [8]. The use of rapid methods applied to scoping reviews has been documented as an increasing trend in big umbrella reviews due to the efficiency gain [9,10]. We followed the PRISMA (Preferred Reporting Items for Systematic Reviews and Meta-Analyses) extension for scoping reviews for the reporting of items relevant to this review. This review did not quality appraise the included studies as this was considered out of scope for the objectives of this project. A preagreed protocol outlining the methods and objectives of this rapid scoping review was registered in the Open Science Framework in June 2024 [7].

### Inclusion Criteria

To outline the inclusion criteria for this rapid scoping review, we followed the Cochrane protocol guide. The review was structured based on the SDMO framework (study type, data, methods, outcomes) [11]. Studies eligible for inclusion needed to meet the following criteria: (1) any type of study or conference abstract in English language, (2) data related to the health care technology were reported, (3) methods and tools used for the analysis were mentioned, and (4) all reported outcomes were considered for inclusion although they were not deemed mandatory for the inclusion of studies in this review in line with the recommendation from Higgins et al [11].

### Search Strategy

Following the recommendation for rapid searches, a targeted search strategy was devised and run in Embase (Ovid), which contains MEDLINE, by an experienced information specialist and checked against the PRESS checklist [12]. Our search strategy consisted of subject heading terms in combination with text words in title and abstract. Given the specificity of the topic and to maximize the sensitivity of our searches, we focused on the Method concept (Patent) from the SDMO framework outlined above. This concept was searched in combination with terms for the different types of studies such as “trend,” “analysis,” “landscape,” “mining” or more broadly “research.” This search was then translated and run in 2 more databases selected for their comprehensive coverage of technological and scientific topics: IEEE Xplore digital library and Web of Science. We searched in the title, abstract, and keyword fields. Time limits were imposed to retrieve records published since 2020 to capture the most contemporary tools and sources. Due to the lack of current guidelines for the application of time limits in searches for rapid reviews, this was a pragmatic decision informed by the recently updated Cochrane Rapid Review Methods guidance and agreed with the information specialist [8]. This approach ensured that our review remained focused on the latest developments and advancements in the field, providing the most relevant and up-to-date information. No language limit or any other limits were used. Records were downloaded into Endnote 20 (Clarivate Analytics) for deduplication. Full search strategies with results are provided in Multimedia Appendix 1.

### Data Collection

Deduplicated results were single screened at the title and abstract stage by 3 independent reviewers (SGGM, EP, and OE) separately using Rayyan (Qatar Computing Research Institute [QCRI]), a screening software for systematic reviews [13]. Consultation between reviewers was practiced at this stage on multiple occasions. In case of doubt due to ambiguity of titles or lack of clear reporting in abstracts, records were included for full-text screening. At full-text screening, the same 3 reviewers proceeded independently to single screen the full texts for inclusion. One-to-one consultation was exercised regularly throughout this process, and disagreements were resolved by checking with a fourth reviewer.

### Data Extraction

To ensure data extraction followed the recommendations of rapid review methods, an abbreviated data extraction form was developed to meet the agreed objectives of this review and was reported in the published protocol [7]. The original data extraction form was then piloted by the reviewers testing the relevance of data fields deemed for extraction. This step did not result in further alterations to the original data extraction form. Three independent reviewers extracted data, and one independently assessed its quality. We extracted bibliographic details; study-related characteristics such as the type of technologies, health condition, and objectives of the study; methods-related data including the sources searched, the geographical coverage of the sources, the time period searched, and the use, if any, of automated methods and tools.

### Data Synthesis

We used narrative and tabulated methods for summarizing and synthesizing data collected during our data extraction stage. Whenever possible, charts have been used to visually present information relevant to the objectives of this study. We used the National Institute for Health and Care Excellence (NICE) disease classification system [14] to map the 96 studies (75%) [15-110] that reported specific disease indications or population groups. A total of 178 separate topic references throughout the 96 papers were extracted and mapped against the NICE classification. Despite some second-level disease indications (eg, COVID-19) that can be mapped to multiple NICE first-level disease classifications, each mention of disease was only mapped once to avoid inflation of results.

## Results

### Overview

**Figure 1.**
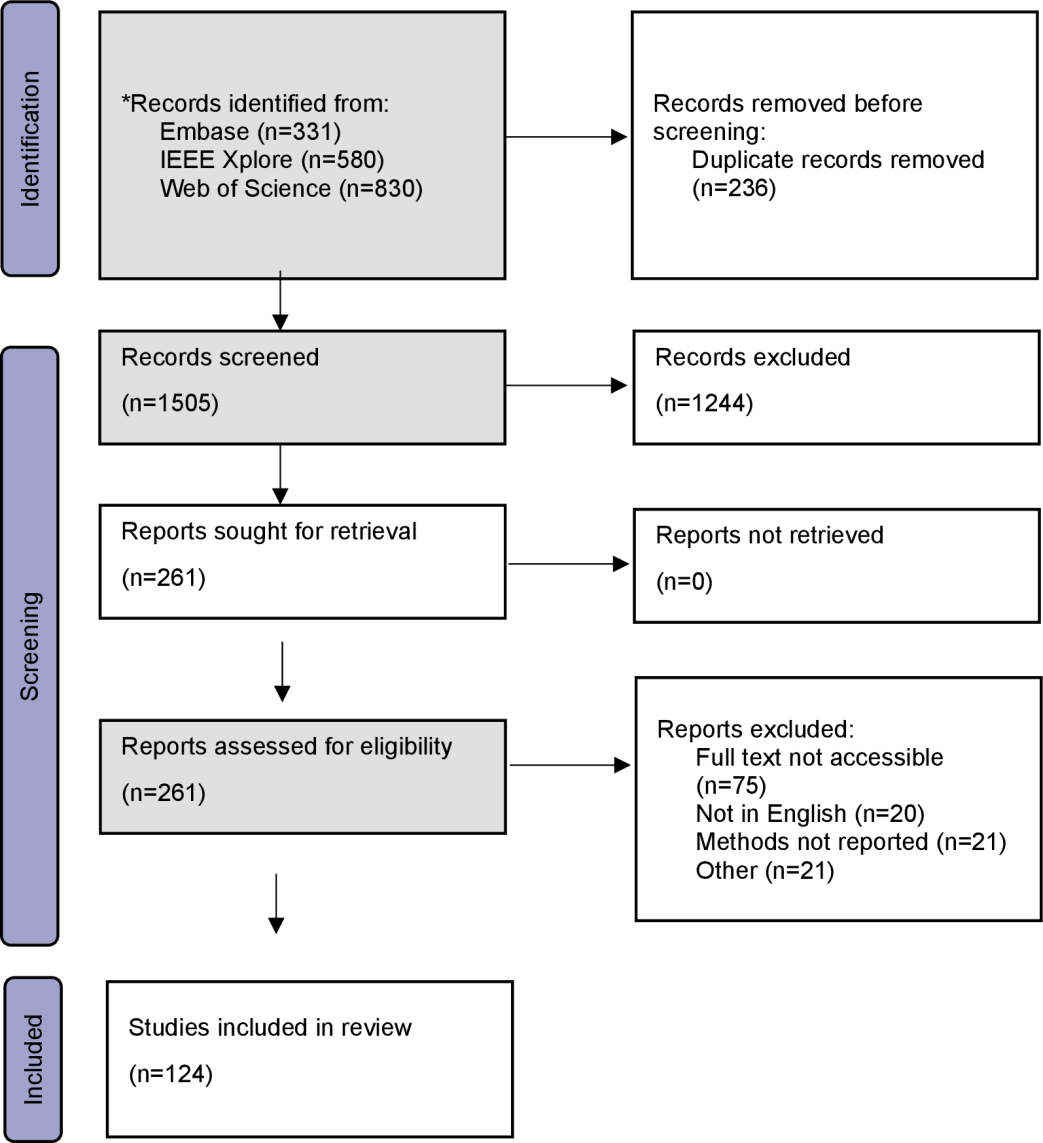
PRISMA flowchart for study selection via searching databases. Gray shading denotes rapid methods, for example, reduced number of databases searched, single screening, and single data extraction. PRISMA: Preferred Reporting Items for Systematic Reviews and Meta-Analyses.

Our searches identified a total of 1741 records. After deduplication, 1505 remained. We sought the full text of 261 records, of which only 124 were considered for inclusion in this review. Figure 1 presents a PRISMA flowchart of the retrieval and selection process for this review.

The selection criteria focused on relevance and the potential to contribute to the review’s objectives. This rigorous process ensured the reliability and comprehensiveness of the findings presented.

### Study Characteristics

From the 124 studies included, 67 (54%) [15-61,111-130] were original research papers, 54 (43.5%) [62-107,131-138] corresponded to reviews, 2 were conference papers [108,109], and 1 short presentation [110]. The majority of sources considered patent databases only (85, 68%) [15-17,111,112,19-26,113,28-32,114,115,35-42,116-118,120,121,43-47,49-51,52-56,124-128,57-60,62,130,65,66,69-72,132,76,77,79-82,87,91,133,94,96,135,98-100,102,104,105,108,110] with the rest of the included studies using a combination of different sources. These 124 studies were published in 94 different journals. *Nature Biotechnology* was the most frequent journal in our dataset (n=9 studies) [15,23,24,52-54,123-125]. A table outlining the characteristics of the included studies is presented in Multimedia Appendix 2 [15-138].

### Research Objectives of Included Studies

The objectives of each paper were extracted verbatim directly from the papers. In order to analyze these, and in line with recommendations on how to analyze and present data from scoping reviews [139], 3 reviewers (SG, EP, and OE) grouped them by common topics. This produced a final list of 9 topics or reasons (Table 1). These were presented and agreed with the rest of the review team. The most common reason reported for the study of patents (62 studies, 50%) [15-17,19,21,24,27,28,111,113,30,31,36,37,39-42,114,116,119,120,46,47,53,54,60,61,65-68,123,126,129-132,72-74,77-79,81,82,87,90,93,133,134,94,97-99,101-103,105,107,108,110] was to investigate trends within specific fields, followed by those aiming to provide recommendations for future research, policy, and strategy development (25 studies, 20%) [20,22,23,25,26,32,33,35,44,121,58,62,127,69,70,76,80,84-86,89,91,92,100,135]. Two other frequent objectives were: the study of specific patented technologies (13 studies, 10%) [18,29,112,34,115,38,48,57,64,71,75,106,109] and the identification of emerging technologies (13 studies, 10%) [18,29,112,34,38,115,48,57,64,71,75,106,109]. Less frequent objectives included the development of methodological frameworks for patent analysis (4 studies) [56,88,117,122], analysis of global patent trends (3 studies) [50,96,137], cross-sectoral applications of emerging technologies (1 study) [55], summarize recent research in specific fields (1 study) [138], analyze patent application pipeline (1 study) [49], and develop patent research methods (1 study) [118].

**Table 1.** Reasons for conducting a patent research study by themes.

Patent analysis objective	Number of studies
Identify and analyze patent trends	62
Provide future directions	25
Identify emerging technologies	13
Analyze patented technologies	13
Methodological framework	4
Compare global patent trends	3
Cross-sectoral applications	1
Summarize recent research	1
Analyze patent application pipeline	1
Develop patent research methods	1

### Sources of Patent Data

Eighty-five studies [15-17,19-26,28-32,35-42,111-118,120,121,43-47,49-56,124-128,57-60,62,65,66,69-72,76,77,79-82,87,91,94,96,98-100,102,105,108,110,130,132,133,135] used patents alone, whereas 39 studies [18,27,33,34,48,119,122,123,61,63,64,67,68,129,131,73-75,78,83-86,88-90,92,93,95,97,134,101,103,106,107,109,136-138] used various sources that included gray literature, research papers, and clinical trials. Two studies [89,96] did not report which patent database had been used. More than 27% (34/124) of papers described the use of more than 1 patent database. As such, across the 124 papers, there were 47 databases listed. Table 2 presents the distribution of the top 10 named databases across all papers.

**Table 2.** Top 10 patent databases used in the included studies.

Patent database	Number of papers
Derwent Innovation	30
USPTO^a^	24
Espacenet	20
The Lens	20
PatentScope	13
WIPO^b^	13
EPO^c^	10
Google Patents	10
Orbit	6
PatSeer	5

a USPTO: United States Patent and Trademark Office.

b WIPO: World Intellectual Property Office.

c EPO: European Patent Office.

### Time Horizons of Patent Scans

Of the 124 included papers, 44 (35.5%) papers [15,22,23,111,36,38,42-45,47-49,52,53,55,56,58,60,61,123,124,126,130,64,66,73,75,76,78-80,83,85,88,91,93,95,99,100,104,108,109,134] did not report the time period searched during patent analysis or gave incomplete timelines with only an end date leaving 80 papers [16-21,24-35,37,39-41,46,50,51,54,57,59,62,63,65,67-72,74,77,81,82,84,86,87,89,90,92,94,96-98,101-103,105-107,110,112-122,125,127-129,131-133,135-138] with completed data on time horizons.

The mean time length covered in those 80 papers was 24.6 years (SD 2.37) [16-21,24-35,112-117,37,39-41,46,50,51,54,118-122,125,127,57,59,62,63,65,67-72,74,77,128,129,131-133,81,82,84,86,87,89,90,92,94,96-98,101-103,105-107,135-138,110]. 7 papers [21,29,41,46,91,106,131] searched a time period of over 50 years with the broadest time period searched being from 1900 to 2019 [140]. Around 19% (15/78) of papers with a reported time horizon searched a period between 3 months and 10 years. Up to 15% (12/78) of papers searched a 20-year period. It was common for authors to backdate searches to the start of the year 2000, although the rationale was not provided.

### Methods and Automated Approaches Used

**Figure 2.**
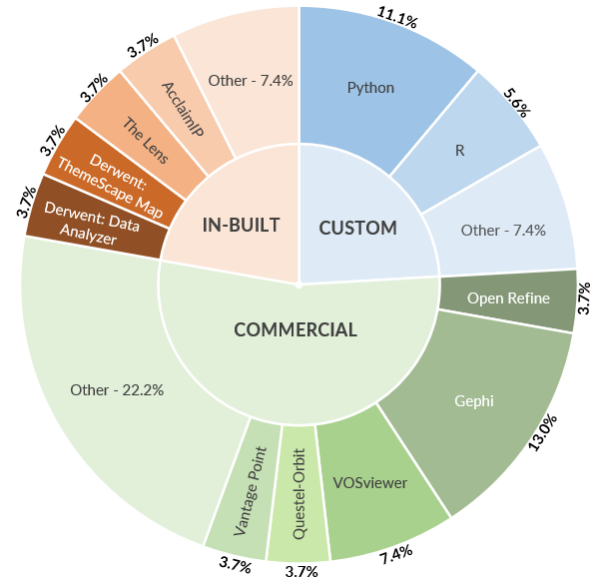
Reported tools used for automated or semiautomated analysis.

Of the 124 included papers, 43 (34.6%) studies [15-35,111-117,62-72,131,132,108,109] reported the use of automated or semiautomated approaches during patent analysis. In these 41 papers, there were 43 unique tools that were used for patent analysis. Figure 2 presents all tools categorized into whether the tool was available as an in-built analysis module by the patent database searched (IN-BUILT), a custom patent analysis script (CUSTOM), or a commercially offered tool (COMMERCIAL).

Some studies mentioned more than 1 tool, a full account of each tool used and their frequency distribution across the 41 studies is available in Table 3. These tools were used mainly in 4 different methods: data mining (understood as the statistical technique of processing raw data in a structured form), text mining (the part of data mining which involves processing of text from documents) [141], data transformation (the process of converting and cleaning raw data from one data source to meet the requirements of its new location) [142], and network visualization (understood as the graphical representations of network devices, network metrics, and data flows) [143]. Network visualization was by far the most popular method, used in 50% of the tools described. The most popular tool for network visualization was the freely available software Gephi (Gephi Consortium), used in 7 separate studies [21-26,113]. Table 3 presents a breakdown of these tools classified by their type and the number of studies that used them.

**Table 3.** Full account of all named tools used for automated or semiautomated approaches for patent analysis from 41 papers that reported their use.

Tool	Number of studies used in	Tool type	Method	Reference
R – tidyverse	2	CUSTOM	Network visualization	[15,16]
R – patentr	1	CUSTOM	Network visualization	[116]
R – cowplot	1	CUSTOM	Network visualization	[16]
R – gridExtra	1	CUSTOM	Network visualization	[16]
R – lubridate	1	CUSTOM	Data transformation	[16]
R – magrittr	1	CUSTOM	Data transformation	[16]
R – linkcomm	1	CUSTOM	Network visualization	[16]
R – trend	1	CUSTOM	Data transformation	[16]
Python – pandas	3	CUSTOM	Data transformation	[16,17,111]
Python – patentpy	2	CUSTOM	Data transformation	[16,116]
Python – seaborn.clustermap	1	CUSTOM	Network visualization	[62]
Python-based Patent Enrichment Tool (PEMT)	1	CUSTOM	Data mining/data transformation	[62]
Python – gensim	1	CUSTOM	Data transformation	[112]
Python – matplotlib	1	CUSTOM	Network visualization	[111]
Latent Dirichlet Allocation algorithm	1	CUSTOM	Text mining	[18]
Word2Vec model	1	CUSTOM	Network visualization	[18]
Term Frequency-Inverse Document Frequency (TF-IDF) model	1	CUSTOM	Text mining	[19]
Generative Topographic Modeling	1	CUSTOM	Network visualization/data transformation	[20]
Gephi	7	COMMERCIAL	Network visualization	[21-26,113]
VOSviewer	4	COMMERCIAL	Network visualization	[27,64,108,131]
Open Refine	2	COMMERCIAL	Data transformation	[64,113]
Questel-Orbit	2	COMMERCIAL	Data mining	[28,132]
Vantage Point	2	COMMERCIAL	Text mining	[29,30]
UCINET 6	1	COMMERCIAL	Network visualization	[111]
Tableau	1	COMMERCIAL	Network visualization	[21]
Patentics	1	COMMERCIAL	Network visualization	[65]
Pajek	1	COMMERCIAL	Network visualization	[22]
Orange-Data Mining	1	COMMERCIAL	Text mining	[66]
Cytoscape	1	COMMERCIAL	Network visualization	[26]
Harzing’s Publish or Perish	1	COMMERCIAL	Data mining	[67]
ITG Insight	1	COMMERCIAL	Text mining/network visualization	[114]
OriginPro	1	COMMERCIAL	Network visualization	[131]
Patsnap	1	COMMERCIAL	Network visualization	[31]
PatentInspiration	1	COMMERCIAL	Network visualization	[68]
NoteExpress	1	COMMERCIAL	Data transformation	[24]
Derwent: ThemeScape Map	2	IN-BUILT	Network visualization	[69,70]
Derwent: Data Analyzer	2	IN-BUILT	Data transformation	[69,108]
Lens	2	IN-BUILT	Data mining	[63,71]
AcclaimIP	1	IN-BUILT	Data mining	[32]
PatentScope	1	IN-BUILT	Text mining	[33]
Derwent: Smart Search	1	IN-BUILT	Data transformation	[115]
Derwent Innovation	1	IN-BUILT	Text mining	[34]
EP full-text search	1	IN-BUILT	Data mining	[72]

### NICE Disease Classification Mapping

Of the 124 papers, 28 (22.6%) [111-116,117-122,123-129,130-132,133-138] did not sufficiently refer to any level of NICE classification. Between the remaining 96 papers [15-110], there were 177 specific mentions of NICE guidance categories, which are detailed below (Table 4). Overall, “Cancer” was the most prevalent NICE topic with 19.4% (19/96) of the 124 included studies reporting cancer patents as either the primary or secondary finding. Of the 16.1% of papers [19,26,28,30,31,33,44,76,84,96,100,104] that reported “Respiratory conditions,” 60% (9/15)^19^,^26^,^30^,^44^,^76^,^84^,^96^,^100^,^104^ of these were specific to “COVID-19” or other coronaviruses.

**Table 4.** Specific mentions of NICE^a^ disease and other guidance topics as a percentage of 178 NICE guidance topics referenced in the results of 94 papers that reported specific NICE topics in their results.

NICE guidance (first level)	Number of mentions	Percentage of papers
Cancer	24	19.4
Infections	20	16.1
Respiratory conditions	19	15.3
Diabetes and other endocrine, nutritional, and metabolic conditions	11	8.9
Injuries, accidents, and wounds	11	8.9
Neurological conditions	11	8.9
Blood and immune system conditions	10	8.1
Cardiovascular conditions	9	7.3
Oral and dental health	9	7.3
Skin conditions	8	6.5
Health and social care delivery	8	6.5
Digestive tract conditions	6	4.8
Mental health, behavioral, and neurodevelopmental disorders	6	4.8
Eye conditions	5	4
Musculoskeletal conditions	5	4
Urological conditions	4	3.2
Chronic and neuropathic pain	2	1.6
Liver conditions	2	1.6
Sleep and sleep conditions	2	1.6
Ear, nose, and throat conditions	1	0.8
Fertility, pregnancy, and childbirth	1	0.8
Lifestyle and well-being	1	0.8
Not reported	28	22.6

a NICE: National Institute for Health and Care Excellence.

## Discussion

### Principal Findings

Our rapid scoping review aimed to explore the recent literature on patent research studies to identify patent sources, tools, and methods used to understand how patent analyses may support horizon scanning methods in the detection of early innovations. To our knowledge, this is the first rapid scoping review focused on methodological approaches for patent research.

This review has identified several key findings that may be used to inform horizon scanning methods. First, our review has revealed an array of methodological approaches, tools, and reasons why patents are being used to discover signs of innovation. We identified 47 different sources for patent data retrieval with some studies using more than one source per study; for example, clinical trials and published or unpublished literature. The compilation of these sources in this review provides an up-to-date published list of patent data sources and insights into how researchers triangulate patent data with information on how, or whether, the innovation is being used in clinical studies and emerging research. This is an important finding for horizon scanning projects in general, and those undertaken by the IO, with particular value for the prioritization of scalable innovation. Patents alone may not be enough to assess the scalability of innovations in health care services such as the UK NHS. Combining patent data with clinical trials, publications, and clinical development analysis offers a more meaningful approach to evaluating technology adoption potential.

Second, this study showed that patent data are mostly used in trend analysis, perhaps due to the accessibility to retrospective global patent data. This finding aligns with what we already know about methods used for scanning emerging technologies and predicting innovations [3,144]. However, this review revealed a less common but notable application of using patent analyses to guide future policy, research, and strategy, similar to the “bottom-up” approach of the European Commission’s Joint Research Centre for detecting weak signals [145]. Most importantly, this approach can also reveal research gaps, thereby aiding in directing future funding and policies to address these gaps, thus contributing to eliminating health inequities and disparities. These findings will contribute to consolidating the IO’s methodological application of patent trend analysis studies to underpin “the push” or “bottom-up approach” to informing UK national future policy, research, or strategy on emerging innovation trends or identified innovation gaps.

Third, from our mapping of disease areas, not surprisingly, cancer, respiratory conditions, and infections were the top 3 topics addressed by the included studies. Considering that this rapid scoping review limited the included studies to only those published between 2020 and 2023, it was expected that COVID or, more broadly, respiratory conditions appeared in our dataset quite prominently. However, the focus on cancer technologies may respond to the growing burden of cancer worldwide and its impact on underserved populations [146]. Likewise, the emphasis on infection topics may respond to the antimicrobial resistance crisis that we are currently facing as well as the challenges posed by the growth of communicable diseases worldwide that continue to be the cause of substantial morbidity and mortality [147]. The alignment with real-world health challenges highlights innovators’ responses and reinforces the need for horizon scanning systems to adopt these methods for studying innovation in major health issues.

Fourth, the analysis showed that tools used for patent data analysis primarily focus on creating graphs and network visualizations, with examples including Gephi [148], VOSViewer (Leiden University) [149], and ThemeScape Map (Derwent Innovation, Clarivate) [150]. Data manipulation tools such as Pandas (Wes McKinney in Python) [151] also played a key role. In addition, Python and R were the most common programming languages used to develop custom tools. Findings from this review highlighted that data transformation and visualization are methods often used for detecting emerging trends. This finding is in line with previously published studies into patent landscaping in the field of life sciences innovation [152]. Since there are no established methodological frameworks for analyzing patent data in horizon scanning, based on these results, it would be reasonable to assume that whenever tools are used for patent data analysis, some level of data transformation (or curation) is needed and therefore this step and the tools needed to undertake it should be part of the repertoire of methods for any horizon scanning practitioner and, more specifically, for the IO’s methods toolkit.

Finally, our review identified a wide range of technologies for which patent analyses were undertaken (Table 3, Multimedia Appendix 2). Due to the short time frame of our analysis, we could not establish a methodological link between analysis methods and technology types. A longer time frame would have yielded more historical data that could have allowed some trend analysis on patent study methods by technology type. Notwithstanding, our results suggest the broad applicability of patent studies to any type of technology.

Our study has some limitations. First, we imposed a short time frame for our bibliographic database searches, which may have meant that we missed some relevant methodological studies. However, this was a pragmatic decision justified by the rapid nature of this review. Given the aims and objectives of our review, which consisted of finding methods and sources for patent landscape analysis studies, we do not think that limiting the search to the last 3 years of publication has biased our methodological conclusions; if anything, it has provided the most up-to-date view on tools and methods. Second, a longer time period would have provided more data allowing us to study the link between technology types and methods providing a more holistic view of patent analysis methods. Third, we used single screening to select studies, so we did not assess interrater agreement. While this practical choice may have led to some relevant studies being missed, we believe it would not have significantly affected our overall conclusions. Finally, our review was not able to ascertain if deduplication of data, a critical step to prevent data inflation and ensure accurate analyses of innovation clusters, was addressed by the included studies [152].

### Conclusions

Our findings highlight a broad range of methodological approaches and tools which may suggest that in the health care technology context, the continuous search for signs of innovation is driving methods and tools development. Our study has shed light on the usefulness and purpose of patent research studies in the context of health care technology innovation, but it has not solved some methodological aspects such as deduplication processes and possible links between technology types and methods. Notwithstanding, when conducting such studies, particular attention should be given to the processes of data manipulation, transformation, and deduplication—especially when integrating multiple data sources—and these methods should be transparently documented and reported in the final output to ensure reproducibility and data quality. Implementing existing guidelines for reporting items for patent landscapes is also strongly recommended [2,153].

The IO aims to provide a systematic approach to horizon scanning across the 3 horizons of health care technology development: emerging, transitional, and imminent. The findings from this study will support the practice of horizon scanning in the emerging technology horizon. In summary, we expect that the findings from this rapid scoping review would be of value to a wide range of horizon scanning practitioners as well as those planning to undertake patent research studies in the field of health care innovation.

## Supplementary material

10.2196/70323 Multimedia Appendix 1 Search strategies.

10.2196/70323 Multimedia Appendix 2 Characteristics of included studies.

10.2196/70323 Checklist 1 PRISMA-ScR checklist.

## Abbreviations

IO: Innovation Observatory
NICE: National Institute for Health and Care Excellence
PRISMA: Preferred Reporting Items for Systematic Reviews and Meta-Analyses
SDMO: study type, data, methods, outcomes
