# Supplementary material for: Horizon Scanning Methods for Health Care Technology Innovation Identification: Rapid Scoping Review of Patent Research Studies
Source: Interact J Med Res. 2025 Sep 11;14:e70323. doi: 10.2196/70323 (PMC12425427; doi:10.2196/70323)
Supplement: Multimedia Appendix 1 [file ijmr-v14-e70323-s001.docx]

Appendix A

Full search strategies used for the identification of patent research studies

**Database:** Embase <1996 to 2024 Week 22>

**URL:** Ovid via University library link

**Data of search:** 06/06/2024

**Number of retrieved records:** 331

**Search strategy:**

1 patent* research.ti,ab,kf,kw. 59

2 patent* analys*.ti,ab,kf,kw. 317

3 patent* landscape.ti,ab,kf,kw. 253

4 (patent* adj3 trend*).ti,ab,kf,kw. 241

5 (patent* adj3 mining).ti,ab,kf,kw. 42

6 or/1-5 812

7 limit 6 to yr="2020 -Current" 331

**Notes:**

Downloaded into Endnote for de-duplication.

**Database: IEEE Xplore Digital library**

**URL: via University library link**

**Data of search: 06/06/2024**

**Number of retrieved records: 580**

**Search strategy:**

("Publication Title": patent research OR "Abstract": patent research OR "Author Keywords": patent research OR "Document Title": patent analysis OR "Abstract": patent analysis OR "Author Keywords": patent analysis OR "Document Title": patent analyses OR "Abstract": patent analyses OR "Author Keywords": patent analyses OR "Publication Title": patent landscape OR "Abstract": patent landscape OR "Author Keywords": patent landscape OR "Publication Title": patent trend OR "Abstract": patent trend OR "Author Keywords": patent trend OR "Publication Title": patent trends OR "Abstract": patent trends OR "Author Keywords": patent trends OR "Publication Title": patent mining OR "Abstract": patent mining OR "Author Keywords": patent mining)

Publication types filter: Conferences, Journals, Early Access Articles

Time limits applied: 2020 - 2024

**Notes:**

All downloaded in csv format.

**Database:** Web of Science

Institute for Scientific Information (2000) Web of science. Philadelphia, PA]: Thomson Reuters.

**URL:** via University link

**Date of search:** 06/06/2024

**Number of retrieved records:** 830

**Search strategy:**

(TS=("patent research") OR TI=("patent research") OR AB=("patent research")) OR (TS=("patent analysis") OR TI=("patent analysis") OR AB=("patent analysis")) OR (TS=("patent analyses") OR TI=("patent analyses") OR AB=("patent analyses")) OR (TS=("patent landscape") OR TI=("patent landscape") OR AB=("patent landscape")) OR (TS=("patent trend") OR TI=("patent trend") OR AB=("patent trend")) OR (TS=("patent trends") OR TI=("patent trends") OR AB=("patent trends")) OR (TS=("patent mining") OR TI=("patent mining") OR AB=("patent mining"))

Publication date limits: 2020, 2021, 2022, 2023

**Notes:**

All exported into Endnote
